# Natural relapses in *vivax *malaria induced by *Anopheles *mosquitoes

**DOI:** 10.1186/1475-2875-7-64

**Published:** 2008-04-22

**Authors:** Lena Huldén, Larry Huldén, Kari Heliövaara

**Affiliations:** 1 Department of Forest Ecology, Faculty of Agriculture and Forestry, University of Helsinki, Finland; 2 Finnish Museum of Natural History, University of Helsinki, Finland

## Abstract

**Background:**

Monthly malaria cases in Finland during 1750–1850 revealed regionally different peaks. The main peak was in late spring in the whole country, but additional peaks occurred in August and December in some regions of Finland. Both primary infections and relapses caused deaths from malaria. The cause and timing of relapses are analysed.

**Methods:**

Monthly data of deaths from malaria in 1750–1850 were successively correlated with mean temperatures of June and July of five years in succession forwards from the current year and through 10 years in succession backwards to identify timing of relapses in *Plasmodium vivax*.

**Results:**

Malaria cases show an increasing correlation with June-July temperatures, with peaks in late summer, midwinter and late spring and then dropped gradually during 2–9 years from the first summer depending on the region. The longest incubation time identified was 8 years and 7 months.

**Conclusion:**

High correlations of June-July temperatures with deaths from malaria in August to September in the same year indicate a close connection to the new generation of hatching *Anopheles *mosquitoes. Because rapid sporogony before October is impossible in Finland, the most plausible explanation is an early induction of relapses of *vivax *malaria by uninfected anophelines. Malaria cases during the winter and the following spring are caused by both primary infections and induced relapses. All subsequent cases represent relapses. It is proposed that the basic relapse patterns in *vivax *malaria are regulated by anophelines. It is also proposed that the *Plasmodium *is enhancing blood sucking of *Anopheles messeae*, which so far has been considered a bad vector.

## Background

It has long been known that *vivax *malaria causes relapses in a variable degree. The malarial relapse including a pre-erythrocytic stage in *Plasmodium vivax *was first outlined in 1948 [1,2], but the true relapse stage, the hypnozoite, was only identified in 1982 [3]. The exact cause of activation of hypnozoites has so far remained unexplained [4]. Regionally different incubation times have been recognized [5-7], suggesting a polymorphic genetic basis for the incubation time [8,9]. In general it seems that *P. vivax *of the temperate zone has a longer incubation time than its tropical counterpart although a long incubation time of tropical malaria is also known [10]. No convincing explanation for the regulation of the incubation time has been presented [11]. In 2004, however, Paul, Diallo and Brey discussed the role of the bites of uninfected mosquitoes in the seasonal transmission of *Plasmodium falciparum *[12].

The northern *P. vivax *disappeared in the 20^th ^century and can only be studied through data in historical sources and archives. The Finnish malaria can be studied according to statistics of deaths from malaria in the parish registers since 1749. These high resolution data are very representative and homogeneous during the time span of 100 years and permits statistical analysis with different parameters. The data also give a detailed insight in the historical distribution of *P. vivax *malaria in northern regions. The mortality in malaria under this period is not biased by the use of cinchona bark or quinine because the ordinary people could not afford them.

June and July temperatures were relevant in influencing the size of the adult *Anopheles *population which hatched in the end of July and August. This had an impact on the malaria situation during the following 12 months [13].

This study aims to explain the underlying cause of relapses and to determine the length of the incubation time of *P. vivax *malaria in Finland.

## Methods

Statistics on malaria deaths in 1750–1850 were collected from the parish registers as described in Huldén *et al *[13]. The closest complete temperature series that cover the time span of the malaria data was taken from Uppsala, Sweden [14]. It has been demonstrated that the use of Swedish temperature records at this level gives a reliable picture of the principal temperature trends in Finland [13].

Data for monthly deaths from malaria in the period 1750 to 1850 were compared with June/July temperatures for the corresponding period and stepwise 10 years backwards in time, 132 months in all (1750–1850, 1749–1849, 1748–1848 etc.). In order to demonstrate the difference between trends and noise the data for monthly deaths from malaria were also compared with June/July temperatures stepwise 5 years forwards in time, 60 months in all (1751–1851 to 1755–1855).

**Figure 1:**
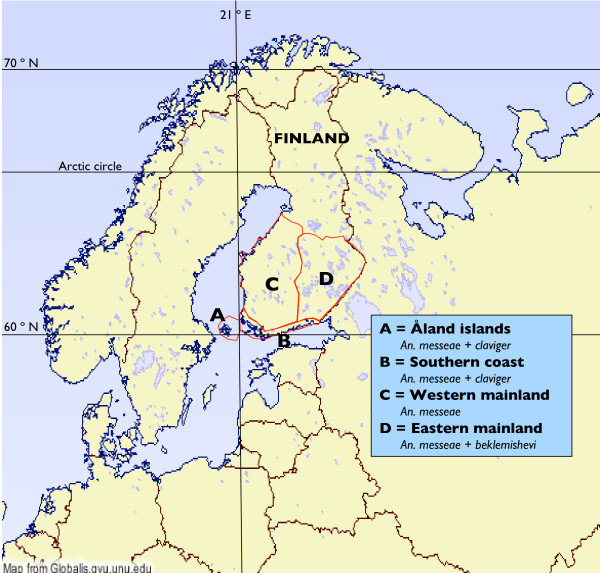
Study regions of malaria deaths in Finland.

The study area was divided into four regions: Åland islands, SW-coast with archipelago, western mainland and eastern mainland (Figure 1). The regions were chosen according to the known distribution patterns of the *Anopheles *species. In the first two regions *Anopheles messeae *is very common with rare finds of *Anopheles claviger*. Åland islands are separated because of a strongly biased gender-related ratio of malaria cases (men/women ca. 40/60%). Men were less exposed to *Anopheles *mosquitoes in the main transmission season in winter/spring because they spent long time in mosquito free conditions when they were out fishing and seal hunting remotely on the sea ice. On the western mainland only *A. messeae *is known, and on the eastern mainland both *A. messeae *and *Anopheles beklemishevi *are common.

## Results

**Figure 2:**
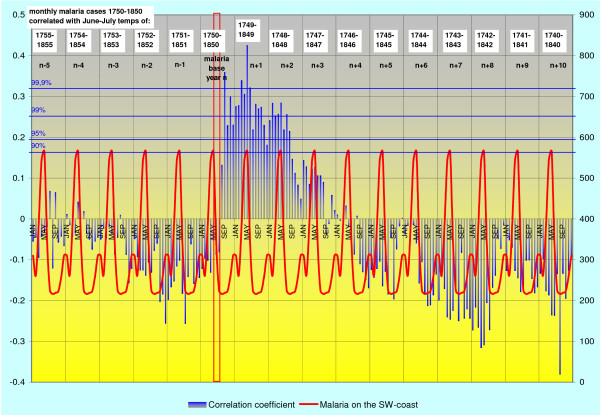
**Monthly deaths from malaria in 1750–1850 on SW-coast and in the archipelago correlated with June-July temperatures (blue bars).** Malaria year is virtually moved from the base year backwards and forwards in time by means of a successive shift of the base year for the temperature records (red vertical bar) in the opposite direction, i.e. forwards and backwards in time. The cumulative monthly death cases from malaria in 1750–1850 (annually repeated red curve), illustrate the monthly frequency of malaria. See also text.

**Figure 3:**
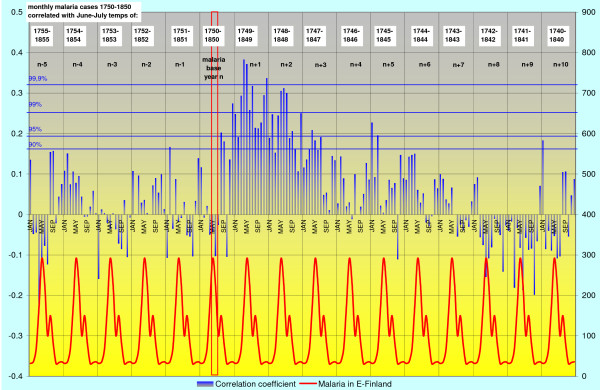
**Monthly deaths from malaria in 1750–1850 on the eastern mainland correlated with June-July temperatures (red vertical bar).** For explanations see Figure 2.

The highest correlation of deaths from malaria with the June-July temperature occurs in April – May during the spring peak of malaria in the following year. The correlation coefficient is then gradually declining during the subsequent years. The regional correlation trends are given for SW-coast and Eastern Finland in Figures 2 and 3.

Most remarkably, there is a sudden increasing correlation between mean temperatures of June – July and deaths from malaria already in August or September during the current year. Because *Anopheles *mosquitoes in Finland are in larval stage in June and July, this high correlation indicates that the new emerging generation of uninfected mosquitoes is somehow involved in the malaria deaths in August and September.

## Discussion

### Induction of relapses

**Figure 4:**
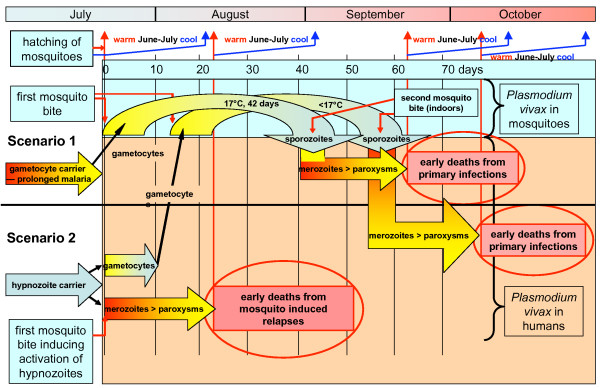
**Theoretical early timing of deaths from malaria in Finland in 1750–1850 in relation to the earliest possible blood meals by new generation of *Anopheles *females.** The earliest deaths from primary infections were possible only in late September and October.

The temperatures in outdoor conditions in Finland are too low for a rapid sporogonic cycle of *P. vivax *to be completed for a new transmission of sporozoites. Nearly all of the primary infections take place indoors during winter and spring [13]. The conclusion is that uninfected anophelines in August and September are inducing an activation of *P. vivax *hypnozoites in humans. As a consequence, relapses in *vivax *malaria are induced by anophelines. The time limits for relapses or infections in relation to timing of deaths from malaria are schematically presented in Figure 4.

It seems probable that only the bites by anophelines can induce a relapse. In Finland the nuisances of mosquitoes outdoors culminates in June and July [15]. An investigation of the man-biting species in early July all over Finland encountered eleven Culicinae species belonging to the genera *Aedes *and *Culiseta*, but no *Anopheles *species [16]. If any of these species could have induced relapses it would have made the monthly correlation pattern of malaria different.

**Figure 5:**
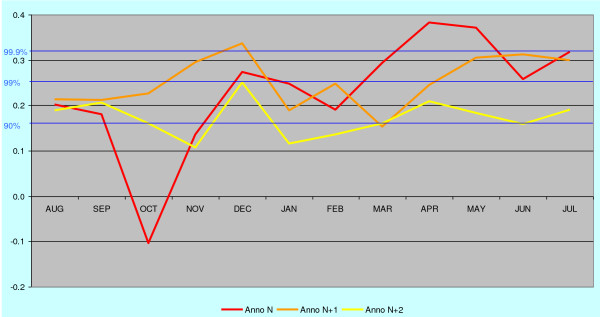
**The annual correlation data for the years n to n+2 from Figure 3 (eastern mainland) shown according to the malaria year defined as August – July.** Primary infections occur only in the winter and spring season of the year n. All subsequent deaths from malaria depend on relapses.

**Figure 6:**
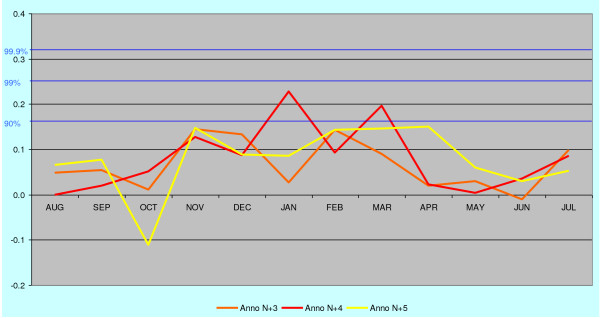
**The annual correlation data for the years n+3 to n+5 from Figure 3 (eastern mainland) shown according to the malaria year defined as August – July.** Midwinter correlations prevail while autumn and spring correlations decline.

**Figure 7:**
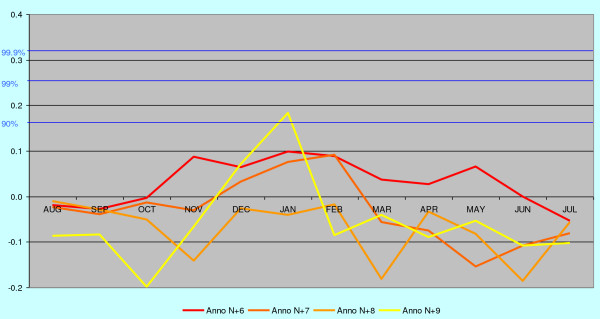
**The annual correlation data for the years n+6 to n+9 from Figure 5 (eastern mainland) shown according to the malaria year defined as August – July.** Traces of midwinter correlations are still visible nearly nine years after the primary infections.

The maximum length of dormancy until the manifested relapse varies between the regions. On the SW-coast and in the western mainland the length of dormancy is less than two years (estimated from the peak of primary infections in April-May). In Åland islands the maximum length is tentatively 3–5 years and in eastern Finland nearly 9 years. The monthly correlation trends of malaria during 10 years after primary infection for eastern mainland are for additional clarity extracted in Figures 5, 6 and 7. The malaria year is presented as the time from August to July in the subsequent year. During the first year high correlation to June-July temperatures in August and September must exclusively represent relapses. Correlation peaks during midwinter are probably mostly caused by relapses with occasional primary infections. This is concluded from the fact that few mosquitoes in the heated cottages had the chance to become infected during the first wave of malaria relapses in August-September because most of the household members then slept separated in unheated buildings. Usually in October people were forced to move together in a heated building because of the decreasing temperatures. Infected mosquitoes in the unheated buildings could not then transmit the parasite further. The proportion of infected mosquitoes in the heated building must have been very low in the beginning of October. Only 15% of *Anopheles messeae *take blood before entering overwintering sites in the beginning of September. Only a small number of these may have got gametocytes from relapses caused by other mosquitoes of the same generation, and finally, only very few of these infected individuals could have by chance entered those buildings where humans in October moved for the winter season. The number of primary infections started to increase at an accelerating rate after midwinter culminating in the high correlation peak in April-May. All subsequent peaks in the correlation (although not necessarily all malaria cases) represent exclusively relapses because the overwintering *Anopheles *dies soon after egg-laying in May. In the case of the eastern mainland the correlation trends reveals that the midwinter peak is preserved for the longest time.

**Figure 8:**
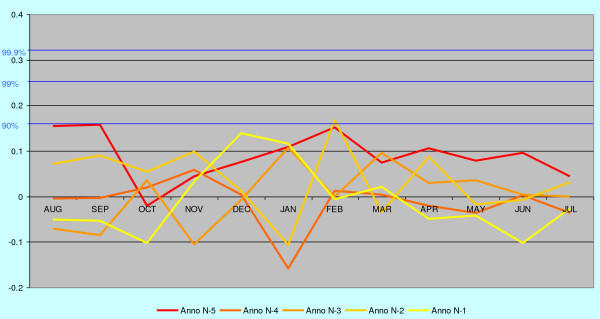
**The annual correlation data for the years n-1 to n-5 from Figure 5 (eastern mainland) shown according to the malaria year defined as August – July.** Correlations with temperatures forwards in time represent random noise and are structurally different from the correlations shown in Figure 8 and.

In order to separate trends from noise, the corresponding trends for malaria death cases in eastern mainland are also correlated against June-July temperatures five years in future, which cannot have any real connections to the deaths from malaria (Figure 8). These five years represent random noise and differ distinctly in structure from the trends 6 to 9 years back in time (Figure 5, 6 and 7), which still cluster in midwinter.

**Figure 9:**
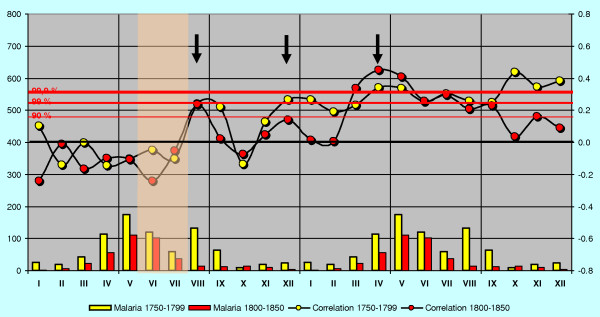
**Correlation for eastern mainland for the years n and n+1 shown separately for 1750–1799 and 1800–1850.** June-July used in correlation shown as a transparent red bar. Correlation peaks approximately indicated with black arrows (both times combined).

In order to check the stability of the regional correlation patterns each regional data series were divided into two periods, 1750–1799 and 1800–1850. These time series can be regarded as two completely independent data sets. The results for two years are presented for eastern mainland in Figure 9. The two correlation series are strikingly similar although the number of malaria death cases may vary between the time series, August in particular. The two time series of Åland islands (not illustrated) deviate slightly from the others, but this is probably due to the exceptional demographic conditions where the male population has changed seasonal means of livelihood. Otherwise the data suggest a robust basis for regionally determined patterns.

### Mosquito/hypnozoite interaction

It is known that the saliva of Anophelines and Culicines has different vasodilators and anticoagulants suggesting independent evolutionary events [17]. When the released sporozoites migrate from the oocysts in the outer wall of the midgut of the mosquito female to the salivary glands, they are detecting and attracted by certain molecules in the structures of the glands. These molecules, however, are not a component of the saliva itself. An ability to detect some component in the saliva must have passed through the sporozoite to the hypnozoite. This information could be imprinted in the sporozoite genome itself or resulting from an interaction between the sporozoite and saliva.

A polymorphic basis of *P. vivax *relapse patterns has been proposed (described as strains or subspecies of *P. vivax*) [8,9]. The current results give some insight into the evolutionary patterns of *P. vivax *biology. It would be a considerable advantage for the *Plasmodium *to be able to alter the timing of gametocyte production in relation to the phenology of the mosquitoes. It is obvious that if the hypnozoite has the capacity to detect blood feeding by the new generation of mosquitoes from humans, the *Plasmodium *can optimize the production of gametocytes when the mosquitoes are abundant. An ability to detect the presence of suitable vectors would also maximize the probabilities of *P. vivax *to adapt to new distant areas with different local vector behaviour. The *Plasmodium *is mainly spreading to the new areas by means of infected humans. In the northern taiga region, the principal time of travel was winter, when adult *Anopheles *mosquitoes were hibernating indoors. As a result the combined behaviour of the vector and human host enhanced cold season transmission and spreading of malaria in northern regions.

The regionally different relapse patterns of malaria, from a maximum of one to two years to a maximum of more than five years, suggest both a western and eastern origin of *P. vivax *in Finland. The prolonged relapse time in eastern Finland may refer to the *P. vivax *strain *hibernans *described from Russia, which gradually dominates towards the taiga region. The western form with a shorter maximum relapse time (one to two years) may refer to the north-west European strain known from Germany, the Netherlands and the British Isles.

If the variation in the relapse time of hypnozoites is a polymorphic character in *P. vivax*, the variation in the relapse time can be explained as a result of regional and seasonal variation in the mosquito vector. Unpredictable seasonality or unpredictable mosquito populations will select for a character that produces hypnozoites with a long relapse time to ensure the survival of *Plasmodium *population. Unpredictability in these conditions increases towards the north and colder regions in the temperate zone and correlates with the increase of long relapse time of *vivax *malaria. The long relapse time in the tropics is less pronounced because the relapse is easily hidden by continuous presence of mosquitoes. Long relapse time up to four years is, however, also known from the tropics [10].

It is assumed that the polymorphic character in *vivax *malaria is defined by the time during which the hypnozoites are resistant to activation. After the resistance time the hypnozoites become sensitive to activation. In natural conditions the sensitive hypnozoites are expected to remain inactive until the new anopheline generation starts to take blood. The anophelines trigger the sensitive hypnozoites to transform into schizonts and to produce merozoites in the human blood. Within a week or two the increasing new generation of anophelines will become infected by gametocytes from human blood. Hypnozoites with longer resistance time would still remain in the human liver until they also become sensitive to activation. Continuous unpredictable conditions (because of climate or other causes) will gradually select for a higher proportion of long incubation time, and may finally lead to regional strains ("forms" or "subspecies") like *hibernans *in which all sporozoites may be transformed into hypnozoites. This simple model explains most observations made on relapses of *vivax *malaria and is fitted for the needs of the *Plasmodium *to survive in variable environmental conditions.

### *Anopheles messeae *as a vector

**Figure 10:**
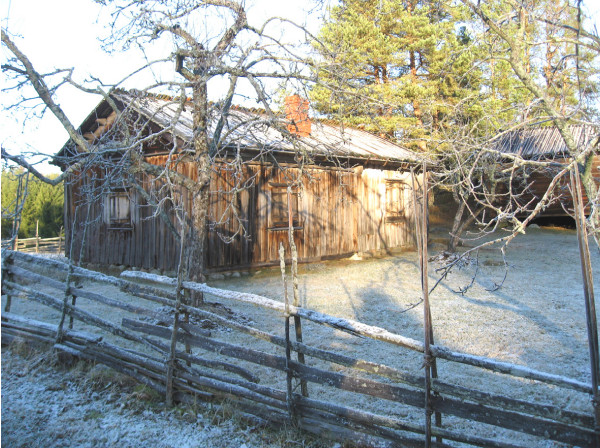
**The croft of Paikkari, Sammatti, in the south of Finland, was a typical building in the countryside.** It was built in 1799–1800 and enlarged by a later owner in 1825. In the more developed parts of Finland chimneys were common, but in the poorer parts of the country the smoke was led out from the building by a hatch in the roof. Originally the croft was a one room (ca 6 × 6 m) log cabin and the birth place of Elias Lönnrot (1802–1884), who collected the Finnish national epos Kalevala. The household consisted of nine persons. The separate sheds were not warmed. Lönnrot's father was a parish-tailor. The family belonged to the stratum under the farmers, but socially represented the crofters, that were the highest group of the non-landowners. The picture is taken in November (2007), the soil is already frozen and the sun is above the horizon for 5 h and 30 min.

*Anopheles messeae *was the only vector covering most of the area where endemic malaria prevailed in Finland. The species has been questioned as a vector in the Netherlands and Sweden. In the Netherlands *A. messeae *increased and *Anopheles atroparvus *declined at the same time as malaria disappeared and it has been assumed that it does not enter heated buildings as often as *A. atroparvus *[18]. In Sweden, it was found that *A. messeae *only marginally take blood before hibernation and that this is not obligatory for the hibernation success [19]. In recent years, however,*A. messeae *has repeatedly been recorded in summer cottages in Finland during the cold season and it has also been observed biting man in the winter season [Kauri Mikkola personal communication 2006]. The Russian research also considers *A. messeae *an important vector in parts of the former Soviet Union [20]. Lewis Hackett observed hibernating *A. messeae *in the beginning of the 1930's. When a mosquito was placed in a warm environment it sucked blood and Hackett concluded that the need for nourishment depended on the conditions in the hibernating place [21]. In 1996, *A. messeae *was identified as the principal vector of resurgent malaria in Russia [22]. The Finnish anophelines entered shelter for hibernation during September and at that time most people still slept in unheated sheds. As a consequence the anophelines entered suitable sheds and buildings at random only some of which later were to be heated. The heating of the crowded traditional log houses in the 18^th ^and 19^th ^century during winter produced very warm and humid conditions which were favourable for semiactive overwintering mosquitoes but which forced the mosquitoes to take extra blood meals to survive. A representative case of late 18^th ^century and early 19^th ^century building is shown in Figure 10.

It is known that the actual number of sporozoites entering humans during blood feeding of an infected mosquito is low although the total production of sporozoites is high [23]. There are indications of enhanced blood feeding with increased number of sporozoites in the salivary glands of *Anopheles punctulatus *and *Anopheles gambiae *[24]. The *Plasmodium *needs to reach as many hosts as possible during the transmission season, which in Finland principally took place during the cold season from midwinter to spring. It is suggested that *P. vivax *similarly enhanced the Finnish vectors (*A. messeae, A. beklemishevi*) to take several blood meals in indoor conditions when the maximum number of sporozoites is available in the salivary glands. If *P. vivax *has the ability to alter the behaviour of the vector, it improves the parasite's capacity to adapt to new regions. Thus the results by Jaenson and Ameneshewa [19] are not in contradiction with the possibility that *A. messeae *can be an effective vector of malaria.

### New interpretation of *Plasmodium falciparum *recrudescence

Hypnozoites are not known in *P. falciparum *and thus falciparum malaria does not have a true relapse. *Falciparum *malaria, however, may persist through non transmission seasons by means of latent trophozoites or schizonts in sequestered red blood cells in the microvascular system, or a process of antigenic variation [25-27]. It is known that in the beginning of the transmission season children under one year get malaria several weeks later than their mothers [12]. It has been suggested that *Anopheles *and/or other genera of mosquitoes cause a sudden increase in infectivity in mothers who have latent schizonts from malaria in the previous transmission season. This "kick start" of malaria was discussed by Paul *et al *[12]. In other words, a mother gets at first a recrudescence while the primary infection in the baby occurs only several weeks later. A new interpretation for this process is proposed. It is assumed that the *Anopheles *mosquitoes actually trigger the schizonts to produce merozoites in the same way as they trigger activation of hypnozoites in vivax malaria. In the case of vivax malaria in Finland, the *Plasmodium *could not afford to respond to any other mosquitoes than *Anopheles *species because of deviating seasonality. Similarly, it would be highly advantageous for *P. falciparum *to produce gametocytes as soon as the vector is becoming abundant. This is the most parsimonous model that explains the observations made on falciparum malaria.

## Conclusion

The relapse mechanism in vivax malaria has long been an enigma to the scientific community. In modern times much of the research on the relapse concerns situations where medication has been in use. It is very probable that medication actually interfere with natural relapses. The Finnish statistical material is quite unique in this respect because practically no effective medication was practiced during 1750–1850. As a consequence natural relapses induced by mosquitoes in the historical statistics can be assessed in Finland.

It is proposed that *P. vivax *has the capacity to detect and alter blood feeding of the *Anopheles *vectors. Increased selection for hypnozoites, the dormant stage, is a response to unpredictable seasonal conditions. By these means *vivax *malaria could spread over a large part of the temperate region and is not limited by climatic conditions.

Ultimately demonstrating a molecule that triggers relapses or recrudescence could create new possibilities for medication of malaria. When hypnozoites can be artificially activated during the nontransmission season the medication will be more effective and further relapses prevented.

## Authors' contributions

LeH drafted the manuscript and collected the historical data. LaH added and/or removed various sections and participated in the design of the study. KH conceived the study and participated in its design. All authors read and approved the final manuscript.
